# Mr. Lyon on Hydrencephalocele

**Published:** 1842-08-27

**Authors:** 


*Hydrencephalocele.*—An interesting case of this rare malformation has been placed on record by Mr. Lyon, one of the surgeons to the Glasgow Royal Infirmary, in a communication published in the "London and Edinburgh Monthly Medical Journal," to which some valuable remarks are attached. A child was born in the year 1841, having tumours on either side of the upper part of the nose, of a glossy, spotted appearance, with numerous small vessels ramifying on the surface. They presented a spongy feel, were diminished by direct pressure, but not by compression of the great arterial vessels. Respiration through the nose perfect, and the palate entire. When the finger was pressed on the tumours, there was an indistinct feeling as if it had entered some cavity. A consultation took place at the infirmary as to the nature of the case: they were at first deemed to be *nævi materni*, from their glossy appearance, vascularity, diminishing under pressure, and resuming their former size when the pressure was removed, their deep red colour, distinct pulsation, and tension while the child was crying. This opinion was abandoned when the tumours were found to be transparent by transmitted light. Then again it was surmised that they were cysts springing from the bones of the face or jaw, connected with the antrum, or else enlarged lacrymal sacs. Their recession and resiliency during and after pressure, their colour, pulsation, &c., controverted the former of these positions, and, together with their situation, showed the second to be erroneous also; while their contents not escaping from the puncta or nostrils during compression, proved they were not caused by dilatation of the lacrymal sacs. The surgeon to whom was due the discovery of their translucency, stated his belief that they were connected with the brain and contained fluid, supplied from between the membranes, the tumours being in his opinion analogous to spina bifida, an hypothesis explaining all the appearances detailed.

The result of this consultation was that one of the tumours should be punctured, an operation that was performed on the one on the right side, when about a drachm of limpid fluid was discharged slowly, and partly from the effect of pressure, which, when exerted on the left side, caused the serum to flow more rapidly, leading to the conclusion that a communication existed between them. The little patient died eight days afterwards, and, on examination of the body, there was found a deficiency of the anterior part of the roof of the ethmoid cells, and of a small portion of the internal angle of the orbitar plate of the frontal bone, through which the tumours, consisting of the membranes of the brain and a part of the anterior cornua of the lateral ventricles, protruded. There was not any osseous partition between the cysts and eyeballs, the ossa plana being wanting. The antra were not involved in the malformation. The ventricles contained about two ounces of serum, and there was effusion of sero-purulent fluid between the dura mater and the arachnoid, and plastic lymph on each of the membranes.

The results of the sectio cadaveris have confirmed Mr. Lyon in his belief that the case was one of hydrencephalocele; and he states his opinion that this malformation, spina bifida, and chronic hydrencephalus, are only three varieties of the same morbid condition, the only difference being one of extent between the first and third, and of locality between them and the second. In this view he holds the treatment adopted to have been incorrectly applied, and thinks puncture of such malformations to be pregnant with highly dangerous consequences. The same treatment is applicable to hydrencephalocele as to spina bifida; and Mr. Lyon alludes to several cases, where persons have reached maturity, notwithstanding the existence of a large spina bifida, and especially to one where a spontaneous cure was effected.

The similarity between hydrencephalocele and spina bifida, he says, is still closer, if we allow the correctness of the idea, maintained by many anatomists, that the bones of the cranium are only the more complete development of the rudimentary parts existing in the vertebræ. In any view of this matter, the similarity of the contents of the skull and spinal canal in organisation, and partially in their functions, the identity of their membranous coverings, and the sameness of their pathological states in the diseases enumerated, authorise us to consider the treatment applicable to one of them as equally proper for the others. This treatment, in Mr. Lyon's opinion, is graduated and equal pressure, applied not merely to the protrusion, but over the whole head, as he believes the tumour to be merely indicative of chronic hydrencephalus.—*Prov. Med. Journ.* June 25, 1842.

